# Deep learning for detecting early gastric cancer with white-light endoscopy: a systematic review and meta-analysis

**DOI:** 10.3389/frai.2026.1734591

**Published:** 2026-01-29

**Authors:** Jixiang Liu, Danyan Li, Yudi Zhuo, Shengsheng Zhang

**Affiliations:** Department of Gastroenterology, Beijing Traditional Chinese Medicine Hospital, Capital Medical University, Beijing, China

**Keywords:** artificial intelligence, deep learning, detection, early gastric cancer, endoscopy

## Abstract

**Background and objectives:**

The aim of this study is to evaluate the performance of DL algorithms in diagnosing early gastric cancer (EGC) using white light endoscopic images.

**Methods:**

A systematic literature search was conducted in PubMed, Embase, Cochrane Library, and Web of Science up to July 25, 2025. Sensitivity and specificity were pooled for internal and external validation sets. The comparison between DL algorithms and expert endoscopists was performed using paired forest plots. Meta-regression was used to identify sources of heterogeneity.

**Results:**

In the internal validation, 15 studies comprising 37,037 images (range: 433–9,650) were included. Pooled sensitivity and specificity were 0.91 (95% CI: 0.82–0.95) and 0.93 (95% CI: 0.87–0.97), respectively. Meta-regression showed that heterogeneity in sensitivity and specificity was significantly associated with training dataset size. For external validation, 4 studies with 3,579 images (range: 200–1,514) were included, yielding pooled sensitivity and specificity of 0.82 (95% CI: 0.61–0.93) and 0.83 (95% CI: 0.74–0.90), respectively. No significant difference was observed between deep learning models and expert endoscopists in diagnostic sensitivity and specificity.

**Conclusion:**

Deep learning algorithms exhibit high diagnostic performance in detecting early gastric cancer using white-light endoscopy. The diagnostic accuracy of DL models is comparable to that of expert endoscopists, supporting their potential role as a clinical decision-support tool.

**Systematic review registration:**

https://www.crd.york.ac.uk/PROSPERO/view/CRD420251112418, identifier CRD420251112418.

## Introduction

Gastric cancer (GC) is a major global health burden, ranking fifth in incidence and fourth in cancer-related mortality worldwide (Sung et al., 2021). Early gastric cancer (EGC) is defined as adenocarcinoma that infiltrates the mucosa or submucosa of the stomach with or without lymph node metastases (T1, any N), which is associated with a favorable prognosis and a five-year survival rate of approximately 95% (Öhman et al., 1980; GASTRIC (Global Advanced/Adjuvant Stomach Tumor Research International Collaboration) Group et al., 2013; Katai et al., 2018; Yang et al., 2021). Consequently, early detection of EGC is critical for improving patient clinical outcomes.

Upper gastrointestinal endoscopy has been established as the gold standard for the diagnosis of EGC (Machlowska et al., 2020). Among its various imaging modalities, white-light endoscopy remains the preferred technique in routine clinical practice due to its widespread availability and ease of use (Nagula et al., 2024). Evidence from South Korea has demonstrated that screening upper gastrointestinal endoscopy has significantly increased the detection of EGC and reduced mortality by approximately 50% (OR = 0.53, 95% CI: 0.51–0.56) (Jun et al., 2017; Arnold et al., 2020). However, EGC lesions often present with subtle mucosal changes, such as microsurface architectural disruption and color irregularities, making their detection challenging under standard white-light endoscopy during routine screening (Zhang et al., 2011; Liu et al., 2023). As a result, the accuracy of EGC detection is highly dependent on endoscopist expertise, resulting in variability in diagnostic performance. Indeed, previous studies have shown that senior endoscopists with more than 10 years of experience achieved significantly higher diagnostic sensitivity in detecting EGC compared to junior endoscopists with only 2–3 years of training (Tang et al., 2020; Yuan et al., 2022).

To address the aforementioned challenges, deep learning (DL)-based artificial intelligence (AI) has been increasingly applied to medical imaging, showing substantial promise in improving diagnostic sensitivity and specificity (Esteva et al., 2019; Gandhi et al., 2025c). Compared to traditional machine learning, DL algorithms possess several advantages. First, they possess the ability to perform feature self-learning from medical image datasets, eliminating the need for manual feature extraction and avoiding potential performance degradation caused by inaccurate or inconsistent segmentation. Second, they can be trained in an end-to-end manner, mapping raw images to diagnostic outputs while jointly optimizing all components of the network (Baldominos et al., 2019; Wang et al., 2019b; Zhou Z. et al., 2023). In recent years, DL algorithms have been widely investigated in the field of pathological image analysis. Numerous studies have consistently demonstrated high diagnostic accuracy in tumor detection across multiple cancer types, including breast, lung, and colorectal cancers, as well as glioma (Wang et al., 2019a; Im et al., 2021; Li et al., 2022, 2025; Thalakottor et al., 2023). In the diagnosis of EGC using endoscopic images, a previous meta-analysis found that conventional AI achieved a sensitivity of 86% and a specificity of 90%, demonstrating diagnostic accuracy comparable to that of experienced endoscopists (Chen P.-C. et al., 2022). However, the aforementioned meta-analysis included a limited number of studies and did not specifically evaluate the performance of deep learning algorithms in detecting EGC under white-light endoscopy.

Therefore, this systematic review synthesizes the latest developments and analyzes the diagnostic performance of DL algorithms on white-light endoscopy image datasets in EGC diagnosis. Meanwhile, our study further compared the diagnostic performance for EGC between DL algorithms and expert endoscopists. The findings will provide evidence-based support for the clinical translation of DL algorithms in upper gastrointestinal endoscopy for EGC.

## Methods

This meta-analysis was conducted in full compliance with the Preferred Reporting Items for Systematic Reviews and Meta-Analyses of Diagnostic Test Accuracy (PRISMA-DTA) guidelines (Supplementary Table 1) (Mdf et al., 2018). Additionally, the study protocol has been registered in the PROSPERO database (CRD420251112418).

### Search strategy

We conducted a systematic literature search using the PubMed, Embase, Cochrane, and Web of Science databases, with the search completed on July 25, 2025. The search strategy involved three groups of keywords: AI-related terms (e.g., artificial intelligence, deep learning), examination-related terms (e.g., endoscopes, gastroscopy), and disease-related terms (e.g., stomach neoplasms, gastric cancer). Both free-text keywords and Medical Subject Headings (MeSH) terms were used to ensure precision. Detailed search strategies are available in Supplementary Table 2. Additionally, the references of included studies were reviewed to identify additional relevant literature.

### Inclusion and exclusion criteria

The studies were systematically selected according to the PITROS framework to ensure methodological clarity and reporting transparency. Participants (P): The participants in this study are patients diagnosed with EGC based on pathological examination. Index test (I): The index test involved the application of DL algorithms to analyze white-light endoscopic images for the automated detection of EGC. Target condition (T): The target condition was the presence of EGC. Diagnosis was based on histopathology, with patients categorized as EGC-positive or EGC-negative accordingly. Outcomes (O): The primary outcomes include sensitivity and specificity for the diagnosis of EGC. Secondary outcomes included a comparative assessment of sensitivity and specificity between DL algorithms and expert endoscopists in the diagnosis of EGC. Setting (S): The study setting includes retrospective or prospective data sources, covering public databases or local hospitals.

Exclusion criteria included studies on animals, non-original articles (e.g., reviews, case reports, meta-analyses, and letters to editors), and non-English publications due to accessibility issues. Furthermore, studies using conventional AI approaches that are unrelated to deep learning algorithms, such as classic machine learning techniques (e.g., support vector machines, logistic regression, and random forests), were excluded. In addition, studies utilizing endoscopic techniques other than white-light endoscopy, such as narrow-band imaging (NBI) or magnifying endoscopy, were excluded.

### Quality assessment

To ensure a rigorous evaluation of the methodological quality of the included studies, we utilized the Quality Assessment of Diagnostic Accuracy Studies-2 (QUADAS-2) tool to assess the risk of bias in predictive modeling (Whiting et al., 2011). The quality evaluation criteria included four domains: patient selection, index test, reference standard, and flow and timing.

### Data extraction

Two independent reviewers (JXL and YDZ) screened the titles and abstracts of the remaining articles to identify potentially eligible studies, with a third reviewer (DYL) acting as an arbitrator to resolve any discrepancies. Extracted data were grouped into three categories: (1) study characteristics (first author, publication year, study design, country of origin, number of centers, diagnostic definition for EGC, and diagnostic algorithm); (2) image dataset composition (number of images in training, internal validation, external validation, DL and endoscopists comparative test set, and tile size); and (3) diagnostic performance outcomes (raw numbers of true positives, false positives, true negatives, and false negatives). For studies lacking information necessary for meta-analysis, we contacted the corresponding authors by email to request the missing data.

### Outcome measures

The primary outcome measures were sensitivity and specificity for internal and external validation sets. Sensitivity, also known as recall or the true positive rate, measures the probability of correctly identifying true EGC cases and is calculated as true positive (TP)/(TP + false negative (FN)). Specificity, or the true negative rate, reflects the probability of correctly identifying non-EGC cases and is calculated as true negative (TN)/(TN + false positive (FP)). For studies comparing the performance of endoscopists and DL algorithms in diagnosing EGC, the diagnostic data of expert endoscopists and DL algorithms will be extracted and entered.

### Statistical analysis

This study employed a bivariate random-effects model to perform the meta-analysis, which jointly pools sensitivity and specificity while accounting for their inherent negative correlation. This model was used to assess the diagnostic performance of deep learning for EGC detection on white-light endoscopy images and to generate a hierarchical summary receiver operating characteristic (HSROC) curve. Sensitivity and specificity were pooled separately for internal and external validation sets. Forest plots visually presented the study-level and pooled estimates, while the SROC curve provided an overall summary with a 95% confidence region and a 95% prediction region. The between-study variance for logit-transformed sensitivity and specificity was quantified using the tau^2^ (*τ*^2^) statistic.

Heterogeneity across studies was evaluated using Higgins’ *I*^2^ statistic, with *I*^2^ values of 25, 50, and 75% indicating low, moderate, and high heterogeneity, respectively (Huedo-Medina et al., 2006). Meta-regression analyses were conducted to identify sources of significant heterogeneity (*I*^2^ > 50%) (van Houwelingen et al., 2002). Meta-regression variables included the number of centers (single or multiple), size of the training dataset (large-scale public datasets or small-scale institutional datasets), validation method (with or without cross-validation), tile size (≤448 × 448 or >448 × 448), and risk of bias in patient selection (high risk or low risk). Potential publication bias was assessed using Deeks’ funnel plot asymmetry test. Furthermore, for comparative assessment of diagnostic performance, sensitivity and specificity were independently pooled for deep learning models and expert endoscopists. Paired forest plots were generated to facilitate direct, visual comparison of sensitivity and specificity across the two groups. Statistical analyses were performed using the Midas package in Stata (version 15.1) and the meta package in R, while risk of bias assessment was conducted with RevMan 5.4 from the Cochrane Collaboration. All statistical tests were two-sided, with *p* < 0.05 considered statistically significant, and results were reported with 95% confidence intervals.

## Results

### Study selection

**Figure 1:**
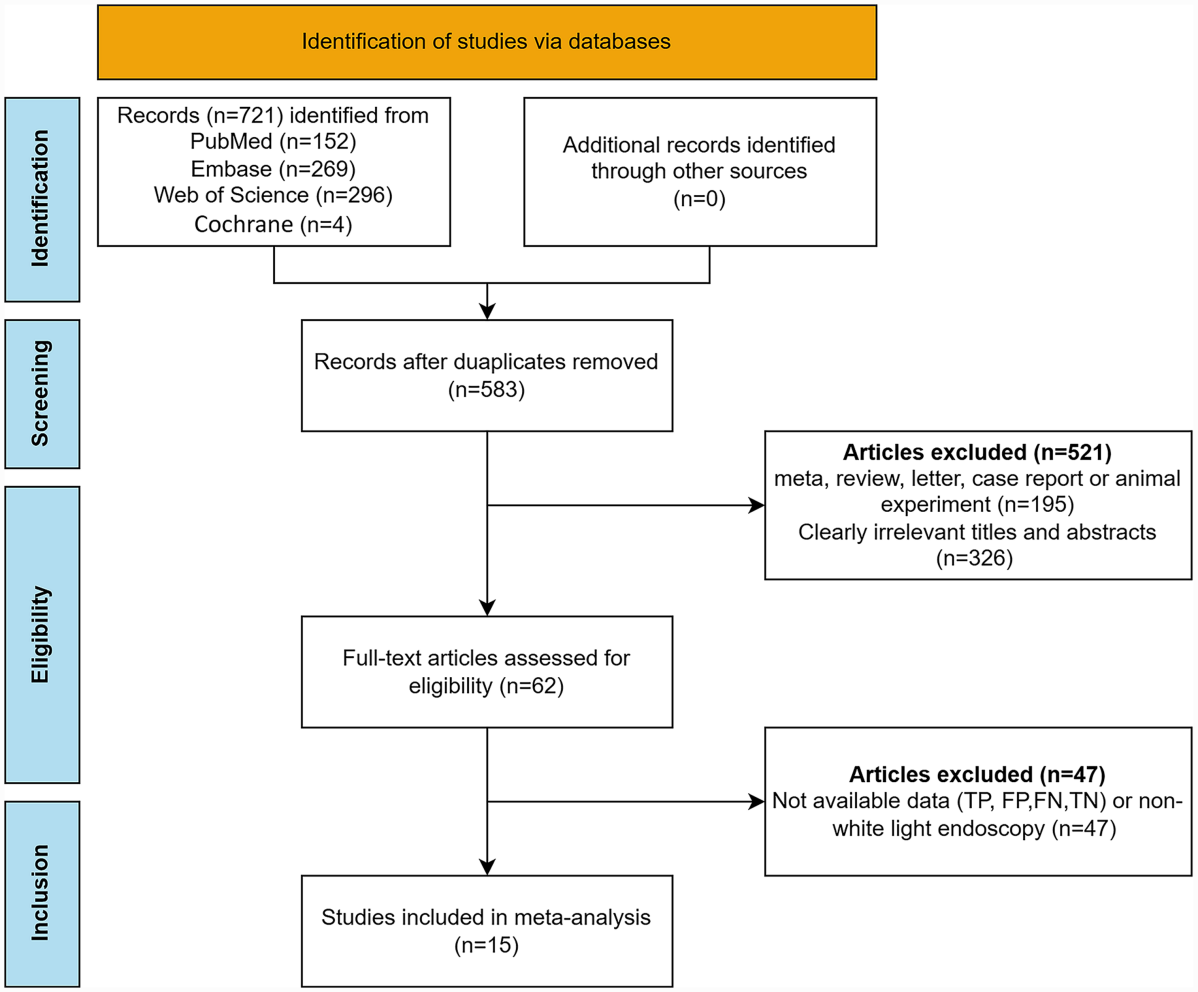
PRISMA flow diagram illustrating the study selection process.

The initial database search identified 721 potentially relevant articles. After removing 138 duplicate records, 583 unique articles underwent preliminary screening. Application of the predefined inclusion criteria led to the exclusion of 521 articles. Subsequently, a comprehensive full-text assessment resulted in the further exclusion of 47 studies due to insufficient or incomplete diagnostic data (TP, FP, FN, TN) or the use of non-white-light endoscopy techniques. Ultimately, 15 studies meeting the eligibility criteria were included in the meta-analysis to evaluate the diagnostic performance of DL algorithms (Sakai et al., 2018; Cho et al., 2019; Tang et al., 2020; Zhang et al., 2021; Teramoto et al., 2022; Yuan et al., 2022; Takemoto et al., 2023; Dong et al., 2023; Zhang et al., 2023; Zhou B. et al., 2023; Chang et al., 2024; Gong et al., 2024; Zhang et al., 2024; Ul Haq et al., 2024; Feng et al., 2025). The literature selection process was summarized using a PRISMA (Preferred Reporting Items for Systematic Reviews and Meta-Analyses) flow diagram, presented in Figure 1.

### Study description and quality assessment

For internal validation, 15 studies involving 37,037 images (range: 433–9,650) were included (Sakai et al., 2018; Cho et al., 2019; Tang et al., 2020; Zhang et al., 2021; Teramoto et al., 2022; Yuan et al., 2022; Takemoto et al., 2023; Dong et al., 2023; Zhang et al., 2023; Zhou B. et al., 2023; Chang et al., 2024; Gong et al., 2024; Zhang et al., 2024; Ul Haq et al., 2024; Feng et al., 2025); for external validation, 4 studies with 3,579 images (range: 200–1,514) were included (Cho et al., 2019; Tang et al., 2020; Yang et al., 2021; Dong et al., 2023; Gong et al., 2024). The studies were published between 2018 and 2025. Regarding study design, 14 studies were retrospective, whereas only one study was prospective in its external validation cohort (Cho et al., 2019). Only two studies utilized large-scale public datasets for training, while the remaining studies were trained using small-scale institutional datasets. All DL models employed in the studies were based on convolutional neural networks (CNNs). Study characteristics and diagnostic performance in internal and external validation are summarized in Tables 1, 2 and Supplementary Table 3, respectively. Notably, five studies included comparisons between DL algorithms and endoscopists in diagnostic performance (Cho et al., 2019; Tang et al., 2020; Zhang et al., 2021; Yuan et al., 2022; Takemoto et al., 2023). The diagnostic performance of DL algorithms and endoscopists is presented in Supplementary Table 4.

**Table 1:** Characteristics of the included studies.

Author	Year	Country	Center	Tile size	Specific model	Model type	Number of images (early gastric cancer vs. control)		Endoscopist comparison
							Training	Validation	
Sakai et al.	2018	Japan	Single	224 * 224	GoogLeNet	CNN^a^	9,587 vs. 9,800	NR^b^	No
Cho et al.	2019	Korea	Multiple	1,280 * 640	Inception-Resnet-v2	CNN	919 vs. 3,286	NR	YES
Tang et al.	2020	China	Multiple	416 * 416	Darknet-53	CNN	26,172 vs. 9,651	NR	YES
Zhang et al.	2021	China	Single	NR	ResNet34	CNN	6,139 vs. 15,078	NR	YES
Zhou et al.	2022	China	Single	512 * 512	EfficientDet-D2	CNN	1,390 vs. 2,232	347 vs. 558	No
Yuan et al.	2022	China	Single	640 * 640	YOLO	CNN	2,015 vs. 27,794	NR	YES
Teramoto et al.	2022	Japan	Single	512 * 512	DenseNet-121	CNN	Imagenet database	5-fold cross-validation	No
Takemoto et al.	2023	Japan	Single	224 * 224	GoogLeNet	CNN	534,926 vs. 593,874	10-fold cross-validation	YES
Gong et al.	2023	Korea	Multiple	512 * 431	NR	CNN	1,766 vs. 13,193	221 vs. 1,650	No
Dong et al.	2023	China	Multiple	NR	YOLO-v3 and Resnet-50	CNN	1,933 vs. 1,679	NR	No
Zhang et al.	2023	China	Multiple	NR	Resnet50	CNN	2,070 vs. 7,966	NR	No
Zhang et al.	2024	China	Single	1,080 * 1,080	Faster RCNN	CNN	Private database and public Kvasir-SEG dataset	5-fold cross-validation	No
Chang et al.	2024	Korea	Multiple	NR	YOLO-v5 and EfficientNetB0	CNN	3,920 vs. 5,026	NR	No
Haq et al.	2024	China	Single	224*224	Faster RCNN	CNN	NR	NR	No
Feng et al.	2025	China	Single	448*448	ResNet18	CNN	3,400 vs. 8,400	NR	No

a CNN: convolutional neural network.

b NR: Not Reported.

**Table 2:** Diagnostic performance of the included studies.

Author	Year	Interval validation sets				External validation sets			
		TP	FP	TN	FN	TP	FP	TN	FN
Sakai et al.	2018	3,723	262	4,735	930	NR	NR	NR	NR
Cho et al.	2019	97	88	559	68	13	33	136	18
Tang et al.	2020	3,967	961	4,303	186	678	98	659	79
Zhang et al.	2021	92	74	766	158	NR	NR	NR	NR
Zhou et al.	2022	376	76	722	69	NR	NR	NR	NR
Yuan et al.	2022	177	146	1,124	9	NR	NR	NR	NR
Teramoto et al.	2022	531	0	1,845	1	NR	NR	NR	NR
Takemoto et al.	2023	387	89	307	75	NR	NR	NR	NR
Gong et al.	2023	164	48	1,602	57	165	119	1,104	39
Dong et al.	2023	104	74	244	11	117	101	211	9
Zhang et al.	2023	365	241	1,373	40	NR	NR	NR	NR
Zhang et al.	2024	263	20	247	13	NR	NR	NR	NR
Chang et al.	2024	451	96	1,665	26	NR	NR	NR	NR
Haq et al.	2024	865	21	829	26	NR	NR	NR	NR
Feng et al.	2025	564	68	617	40	NR	NR	NR	NR

TP, true positive; TN, true negative; FP, false positive; FN, false negative; NR, Not Reported.

**Figure 2:**
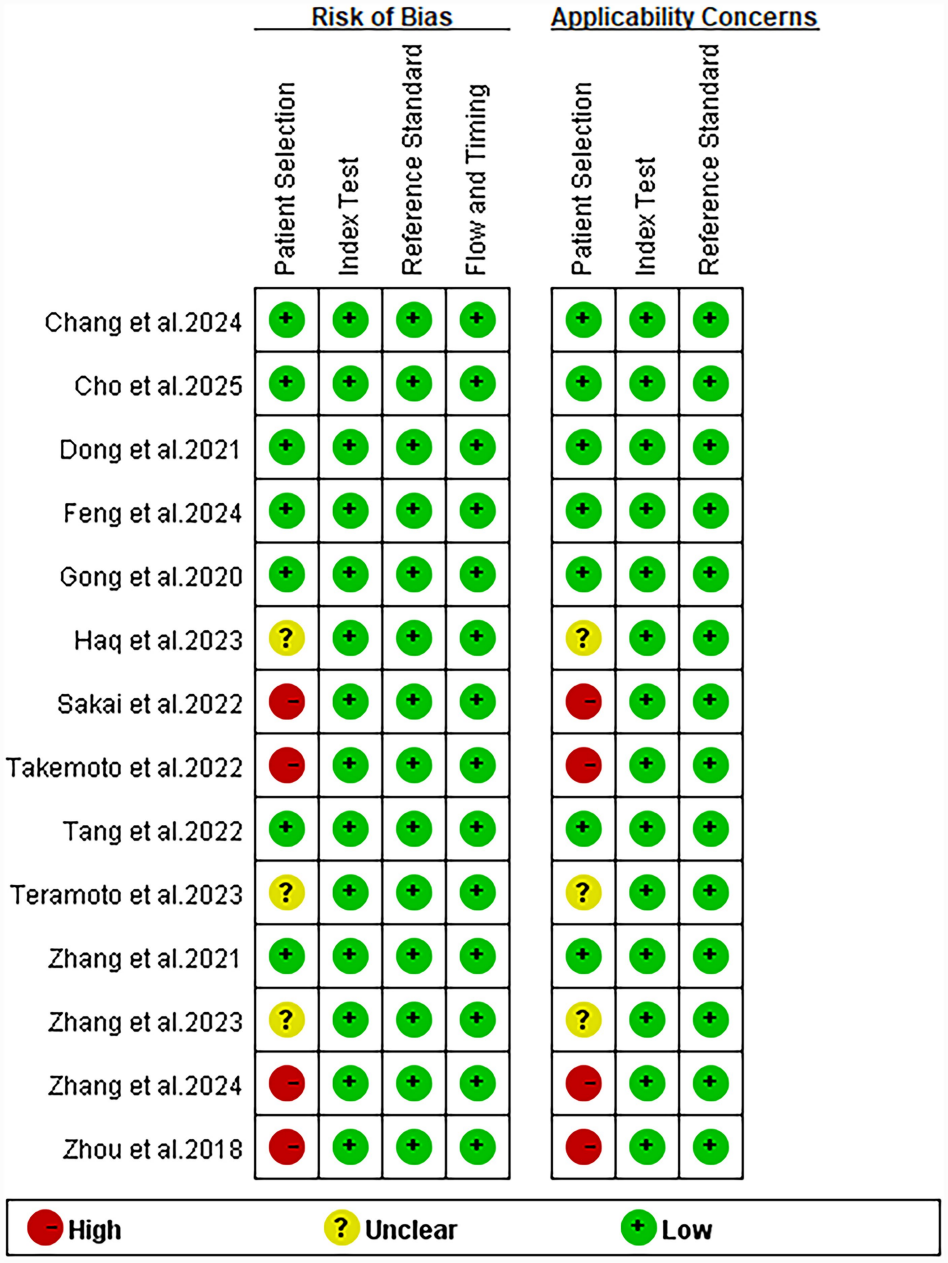
Risk of bias and applicability concerns in the included studies, assessed using the Quality Assessment of Diagnostic Accuracy Studies (QUADAS-2) tool.

The risk of bias, assessed using the revised QUADAS-2 tool, is illustrated in Figure 2. In the patient selection domain, four studies were classified as “high” due to insufficient reporting on patient recruitment (e.g., whether enrollment was conducted consecutively). All studies were deemed to have a low risk of bias in the index test, reference standard, and flow and timing domains.

### Diagnostic performance of deep learning algorithms in the internal validation set for early gastric cancer detection

**Figure 3:**
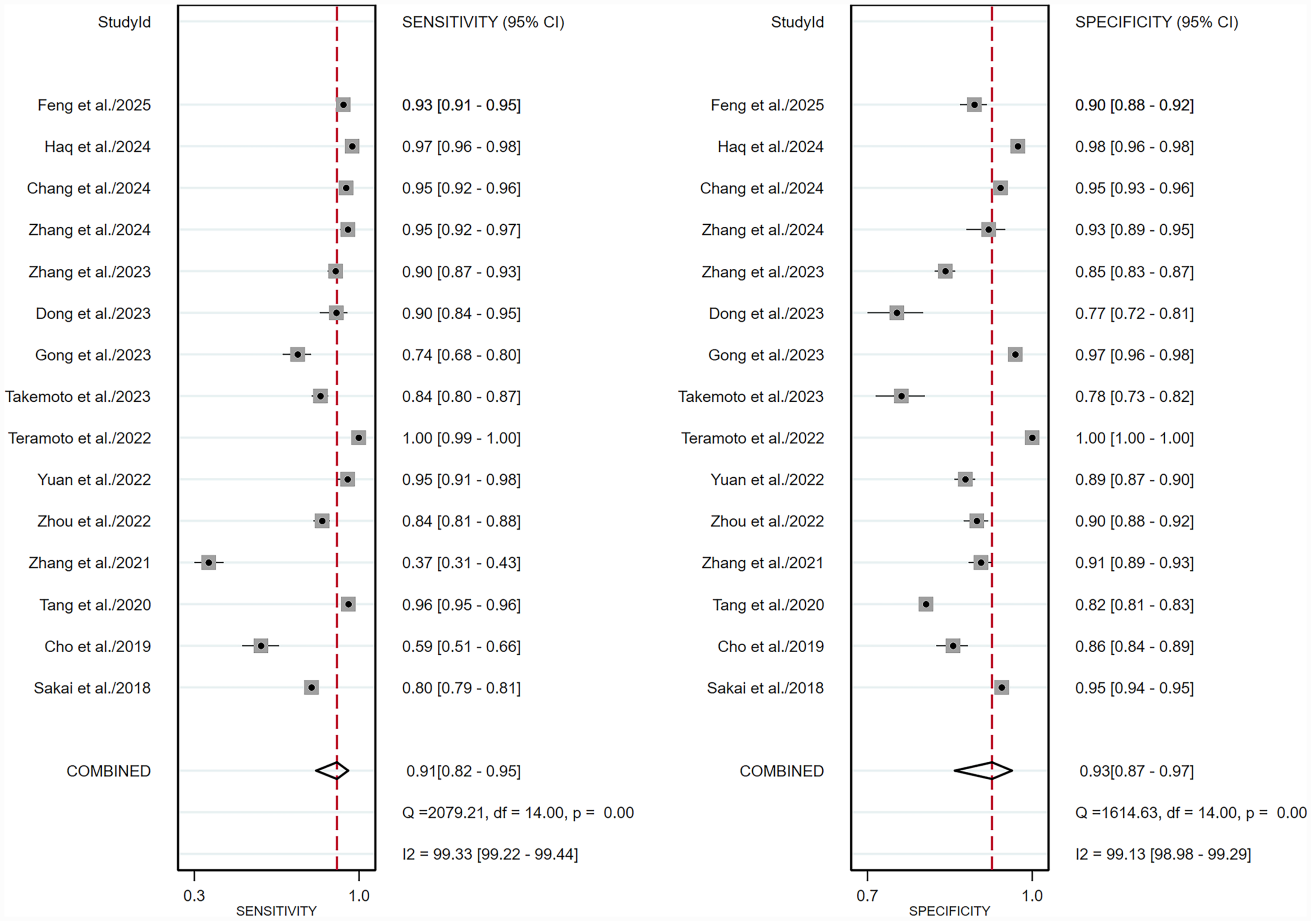
Forest plot of sensitivity and specificity of deep learning algorithms for detecting early gastric cancer (EGC) in the internal validation set. Squares represent individual study estimates, with horizontal lines indicating 95% confidence intervals; the diamond denotes the pooled estimate.

**Figure 4:**
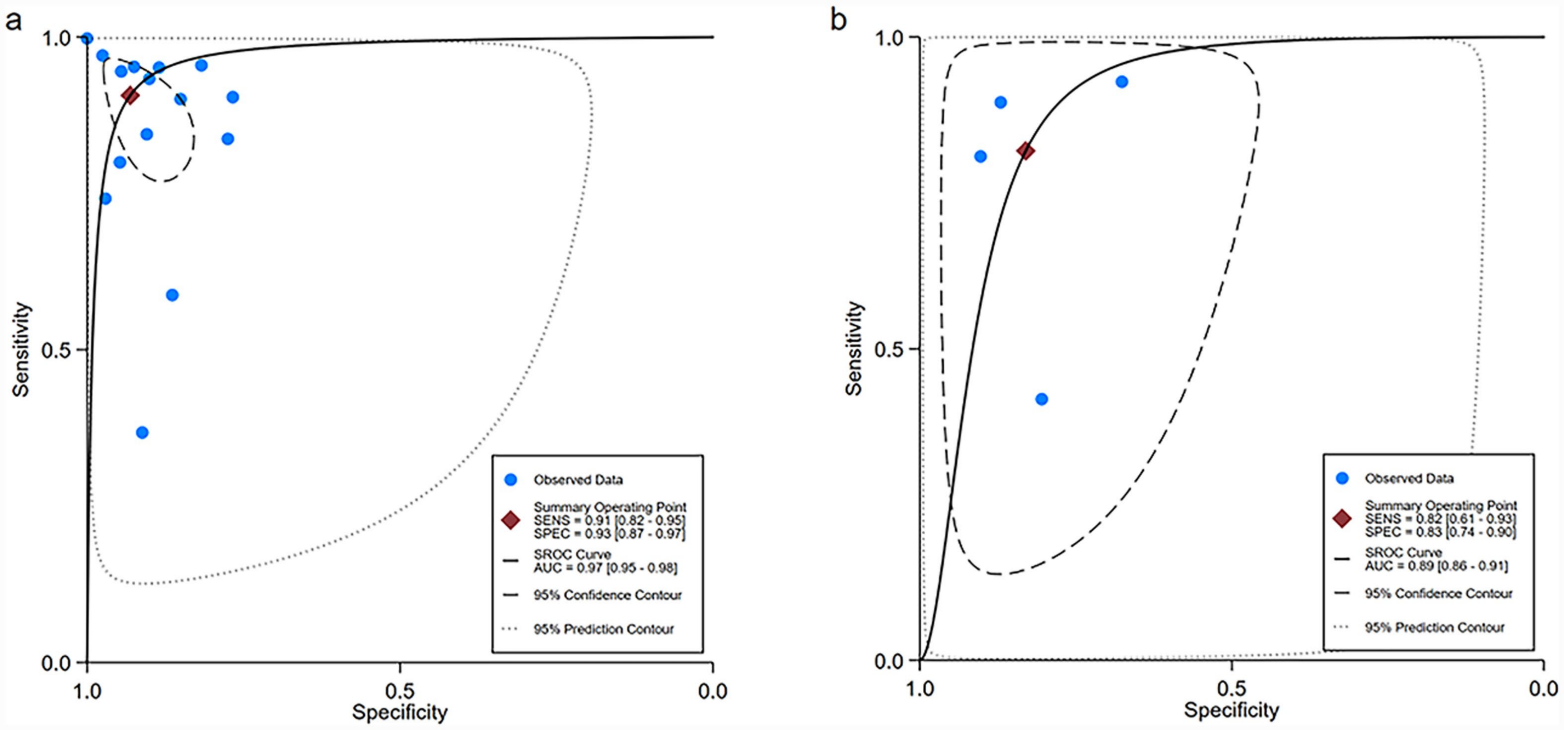
Summary receiver operating characteristic (SROC) curves of deep learning algorithms for detecting early gastric cancer (EGC) in the internal **(a)** and external **(b)** validation sets.

**Figure 5:**
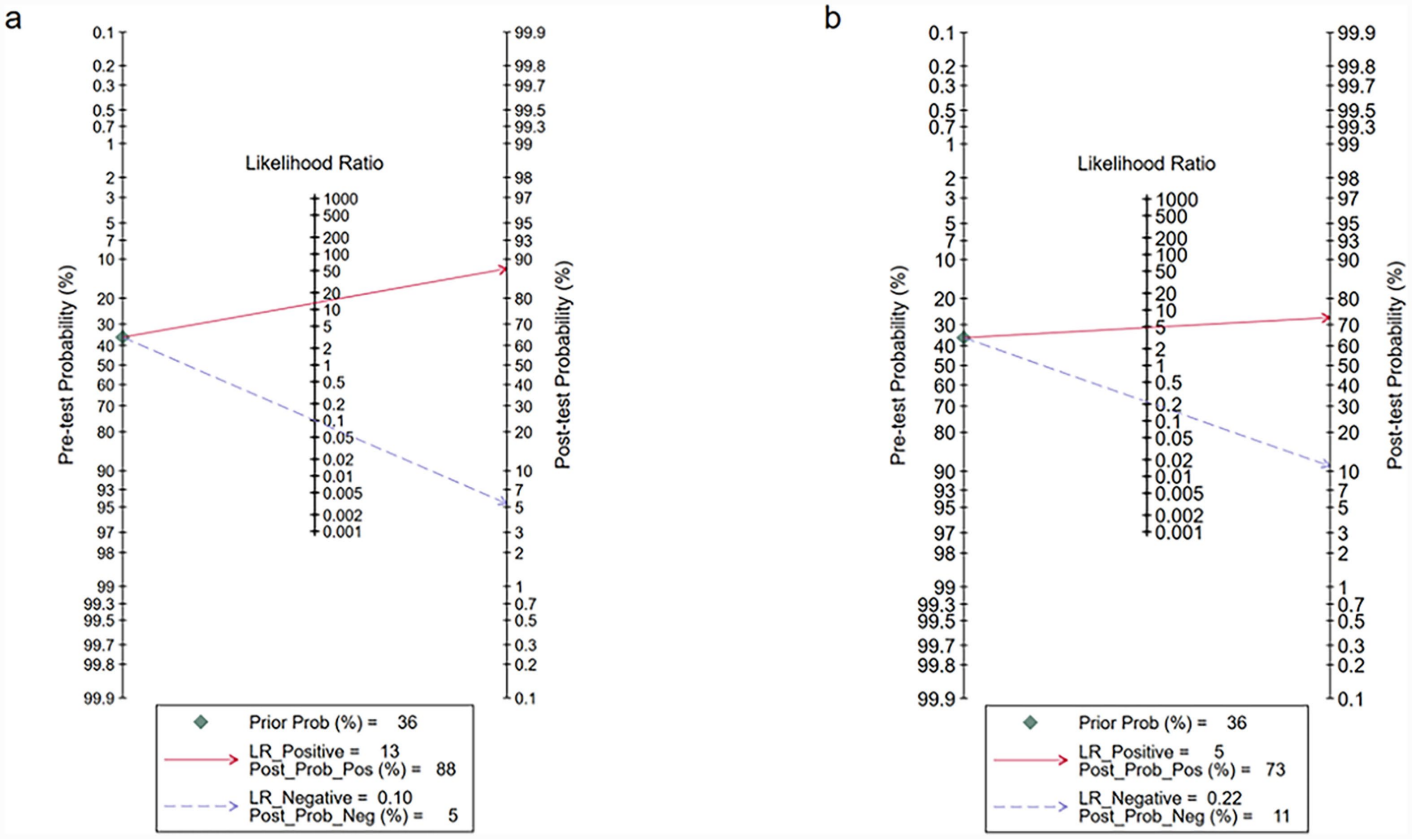
Fagan’s nomogram illustrating the clinical utility of deep learning algorithms for detecting early gastric cancer (EGC) in the internal **(a)** and external **(b)** validation sets.

For the internal validation dataset, DL algorithms based on white endoscopy images achieved a sensitivity of 0.91 (95% CI: 0.82–0.95) and a specificity of 0.93 (95% CI: 0.87–0.97) in detecting EGC patients (Figure 3). The area under curve (AUC) was 0.97 (95% CI: 0.95–0.98) (Figure 4a). With a pre-test probability of 36%, representing the average incidence rate across all studies included in the internal validation dataset, the Fagan nomogram demonstrated a positive likelihood ratio of 88% and a negative likelihood ratio of 5% (Figure 5a).

High heterogeneity was observed in both sensitivity (*I*^2^ = 99.33%, *τ*^2^ = 1.89) and specificity (*I*^2^ = 99.13%, *τ*^2^ = 2.46) within the internal validation dataset. Meta-regression analysis revealed that heterogeneity in both sensitivity and specificity was significantly associated with the size of the training dataset (large-scale public datasets vs. small-scale institutional datasets, *p* < 0.05) and validation method (cross-validation vs. without cross-validation, *p* ≤ 0.05) (Table 3). Level-one out sensitivity analysis did not identify any influential studies or potential sources of heterogeneity (Supplementary Table 5). In addition, after excluding studies with a high risk of bias, the sensitivity was 0.86 (95% CI: 0.72–0.94) and the specificity was 0.90 (95% CI: 0.85–0.93), yielding a summary AUC of 0.94 (95% CI: 0.92–0.96).

**Table 3:** Meta-regression analysis of diagnostic performance of deep learning models for early gastric cancer (EGC) in internal validation cohorts.

Subgroup	Studies, *n*	Sensitivity (95%CI)	Meta-regression *p*-value	Specificity (95%CI)	Meta-regression *p*-value
Center			0.96		1.00
Single-center	9	0.92 (0.85–0.99)		0.95 (0.91–0.99)	
Multi-center	6	0.88 (0.75–1.00)		0.89 (0.79–1.00)	
Training dataset			0.01		0.00
Large-scale public datasets	2	0.99 (0.97–1.00)		0.99 (0.98–1.00)	
Small-scale institutional datasets	13	0.87 (0.80–0.95)		0.91 (0.85–0.96)	
Validation method			0.05		0.09
Cross-validation	3	0.97 (0.93–1.00)		0.98 (0.94–1.00)	
Without cross-validation	12	0.88 (0.79–0.96)		0.91 (0.85–0.97)	
Tile size			0.84		0.33
≤448*448	5	0.92 (0.84–1.00)		0.96 (0.92–1.00)	
>448*448	6	0.92 (0.83–1.00)		0.91 (0.79–1.00)	
Risk of bias in patient selection			0.87		0.99
High	4	0.87 (0.71–1.00)		0.90 (0.78–1.00)	
Unclear or low	11	0.92 (0.85–0.98)		0.94 (0.89–0.99)	
Control group composition			0.87		0.99
Normal mucosa	4	0.87 (0.71–1.00)		0.94 (0.89–0.99)	
Mixed normal and precancerous mucosa	11	0.92 (0.85–0.98)		0.90 (0.78–1.00)	
Year of publication			0.47		0.68
≤2020	3	0.83 (0.61–1.00)		0.89 (0.73–1.00)	
>2020	12	0.92 (0.86–0.98)		0.94 (0.89–0.98)	
DL model types			0.80		0.57
Image classification models	10	0.88 (0.79–0.97)		0.93 (0.87–0.99)	
Lesion detection models	5	0.94 (0.88–1.00)		0.94 (0.86–1.00)	

### Diagnostic performance of deep learning algorithms in the external validation set for early gastric cancer detection

**Figure 6:**
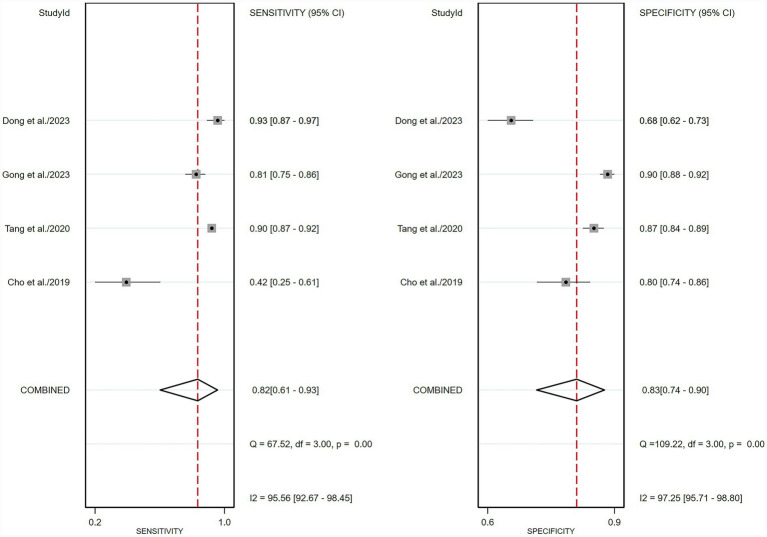
Forest plot of sensitivity and specificity of deep learning algorithms for detecting early gastric cancer (EGC) in the external validation set. Squares represent individual study estimates, with horizontal lines indicating 95% confidence intervals; the diamond denotes the pooled estimate.

For the external validation dataset, DL algorithms based on white endoscopy images achieved a sensitivity of 0.82 (95% CI: 0.61–0.93) and a specificity of 0.83 (95% CI: 0.74–0.90) in detecting EGC patients (Figure 6). The AUC was 0.89 (95% CI: 0.86–0.91) (Figure 4b). With a pre-test probability (prevalence) of 36%, the Fagan nomogram demonstrated a positive post-test probability of 73% and a negative post-test probability of 11% (Figure 5b). High heterogeneity was observed in both sensitivity (*I*^2^ = 95.56%, *τ*^2^ = 1.09) and specificity (*I*^2^ = 97.25%, *τ*^2^ = 0.31) within the external validation dataset. Due to the limited number of included studies, meta-regression analysis was not performed to explore potential sources of heterogeneity.

### Deep learning algorithms versus endoscopists: performance in early gastric cancer detection in the test set

**Figure 7:**
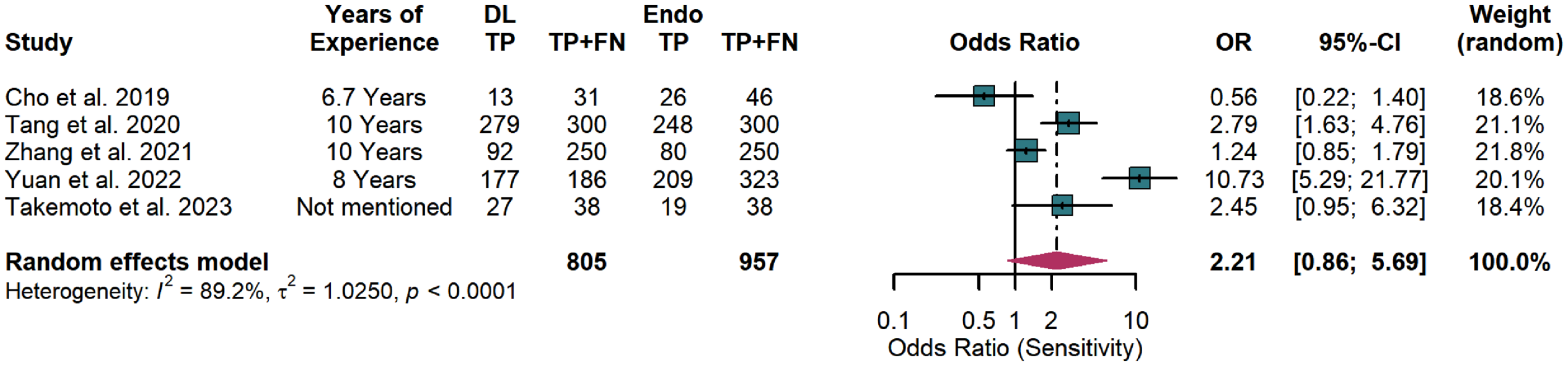
Forest plot comparing the sensitivity of artificial intelligence and endoscopists in detecting early gastric cancer (EGC) in the test set.

In the comparison between the DL model and endoscopists on the test set, substantial heterogeneity was observed in diagnostic sensitivity (*I*^2^ = 89.2%, *p* < 0.0001) (Figure 7). A random-effects model was used for primary analysis, which showed no statistically significant difference between the two groups (pooled OR = 2.21, 95% CI: 0.86–5.69), indicating comparable sensitivity performance.

**Figure 8:**
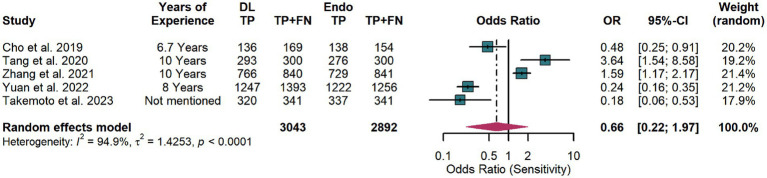
Forest plot comparing the specificity of artificial intelligence and endoscopists in detecting early gastric cancer (EGC) in the test set.

Similarly, for diagnostic specificity, significant heterogeneity was present (*I*^2^ = 94.9%, *p* < 0.0001) (Figure 8). The random-effects model revealed no significant difference between DL and endoscopists (pooled OR = 0.66, 95% CI: 0.22–1.97), suggesting similar specificity performance.

### Publication bias

The Deeks’ funnel plot asymmetry test showed no significant publication bias in the internal validation dataset based on white light endoscopy images for DL (*p* > 0.05) (Supplementary Figure 1). In contrast, the Deeks’ funnel plot asymmetry test revealed significant publication bias in the external validation dataset, which consisted of only four studies utilizing white light endoscopy images (*p* < 0.05; Supplementary Figure 2).

## Discussion

To the best of our knowledge, this is the first meta-analysis to comprehensively evaluate the performance of DL algorithms in diagnosing EGC using white light endoscopic images. The results indicate that DL algorithms exhibit excellent diagnostic performance in the internal validation set, with a sensitivity of 0.91, a specificity of 0.93, and an AUC of 0.97. In the external validation set, the diagnostic sensitivity, specificity, and AUC were 0.82, 0.83, and 0.89, respectively, which were lower than those in the internal validation set. Furthermore, no significant differences were observed between DL algorithms and expert endoscopists in terms of diagnostic sensitivity or specificity. Meta-regression analysis indicates that the sample size of the training dataset contributes to the high heterogeneity in sensitivity and specificity observed in the internal validation sets. In summary, these results suggest that DL algorithms demonstrate good diagnostic performance in detecting EGC using white-light endoscopic images, indicating their potential as a reliable auxiliary diagnostic tool.

Sensitivity and specificity are key metrics for evaluating diagnostic performance. In this study, the DL model demonstrated high sensitivity and specificity in the internal validation set. High sensitivity indicates a low risk of missed diagnosis, facilitating the detection of EGC with atypical morphology or indistinct borders. High specificity reflects a low false-positive rate, conducive to reducing unnecessary biopsy procedures and thereby preventing overdiagnosis and overtreatment. The strong performance observed in the internal validation may be attributed to consistent data preprocessing, standardized image acquisition protocols, and uniform endoscopic imaging conditions (Li et al., 2025). These factors help minimize technical variability, enabling the model to more accurately distinguish EGC from non-EGC findings. However, in the external validation set, both sensitivity and specificity were lower than those observed in the internal validation. This performance decline is likely due to real-world variations across institutions, such as differences in endoscopist expertise, types of endoscopic equipment, and image quality (Campanella et al., 2019). These heterogeneities introduce noise and complexity that the model may not have fully accounted for during training. These findings underscore the importance of standardized data pipelines and the use of diverse, multi-center datasets during model development to improve model generalizability and robustness.

Currently, due to limitations in technical skills and clinical experience, trainee endoscopists exhibit significantly lower sensitivity and specificity in diagnosing EGC compared to expert endoscopists (Ende et al., 2018; Tang et al., 2020; Yuan et al., 2022). This performance gap contributes to instability in clinical endoscopic practice and increases the risk of missed or incorrect diagnoses, especially in primary care hospitals. Previous studies revealed that, with AI assistance, trained novices can produce expert-level lung and cardiac ultrasound images that can be used to assess pathology after a short training session, thereby enhancing access to diagnosis in resource-constrained settings (Narang et al., 2021; Baloescu et al., 2025). In this study, our results demonstrate that DL algorithms achieve sensitivity and specificity comparable to those of expert endoscopists. Therefore, it is reasonable to hypothesize that AI may serve as an effective assistive tool to enhance the sensitivity and specificity of trainee endoscopists in the detection of EGC during white-light endoscopy screening, thereby minimizing the likelihood of missed or incorrect diagnoses and facilitating earlier detection and timely intervention.

In the internal validation of deep-learning algorithms, meta-regression analysis demonstrated that models trained on large-scale public datasets exhibited significantly superior diagnostic sensitivity and specificity compared to those trained on small-scale institutional datasets. This finding indicates that the size of the training dataset may be one of the key factors determining the diagnostic performance of the deep-learning algorithms. Previous studies revealed that merely expanding the size of the training dataset can improve the classification performance of the DL network (Kiryati and Landau, 2021; Pei et al., 2021). However, due to the challenges in acquiring and annotating medical imaging data, particularly in the three-dimensional context of endoscopic examinations, constructing large and high-quality training datasets was difficult (Tajbakhsh et al., 2016; Chen X. et al., 2022). In contrast, public datasets offered a viable pathway to overcome these difficulties. ImageNet is a large-scale hierarchical visual recognition database developed in the United States, comprising 14 million manually labeled images (Kang et al., 2021). Kvasir-SEG is a publicly accessible high-quality gastrointestinal endoscopy dataset originating from Norway, comprising 1,000 images annotated with pixel-level segmentation masks (Jha et al., 2019). Consequently, in this meta-analysis, deep-learning algorithms trained on ImageNet and Kvasir-SEG datasets achieve superior performance in EGC detection. Furthermore, although cross-validation is an important technique for evaluating model robustness, particularly in studies with small datasets, our analysis did not observe a significant influence of cross-validation on heterogeneity within the internal dataset (Aggarwal et al., 2022). Similarly, factors including the number of participating centers, image size, and study quality did not contribute significantly to internal heterogeneity. However, this heterogeneity may stem from other potential factors such as clinical staging of EGC, image quality, and variations in the definition of EGC.

To our knowledge, this is the first meta-analysis specifically evaluating the diagnostic performance of DL algorithms for EGC. In contrast, a prior meta-analysis of 12 studies reported that AI—encompassing both machine learning and DL algorithms—achieved a sensitivity of 0.86 and a specificity of 0.90 in the diagnosis of EGC, values notably lower than the 0.91 and 0.93 observed in this study (Chen P.-C. et al., 2022). This discrepancy may be attributed to differences in algorithmic model selection (DL versus a combination of machine learning and DL). At the algorithmic level, traditional machine learning methods rely on handcrafted feature engineering and exhibit limited generalizability, particularly when applied to complex and heterogeneous medical imaging data (Moawad et al., 2022). In contrast, the DL models evaluated in this study enable end-to-end learning by automatically extracting hierarchical feature representations directly from raw images, thereby achieving enhanced robustness and higher diagnostic accuracy in complex visual recognition tasks (Wang et al., 2019b).

With a pre-test probability of 36%, the Fagan nomogram demonstrated a positive post-test probability of 73% and a negative post-test probability of 11%. This provides a practical tool for clinicians: for a patient with a pre-test suspicion of 36%, a positive result from the DL model would increase the probability of EGC to 73%, warranting a confirmatory biopsy. Conversely, a negative result would lower the probability to 11%, potentially supporting a decision for surveillance rather than immediate intervention, depending on the clinical context. From a clinical implementation perspective, these findings support the role of DL-based systems as decision-support tools rather than standalone diagnostic solutions. Practical deployment would require targeted training for endoscopists on AI-assisted interpretation within endoscopy suites, alongside clearly defined safety workflows to ensure clinician oversight (Olawuyi and Viriri, 2025). Moreover, regulatory approval is a prerequisite for clinical adoption. Similar to AI-based electrocardiogram detection systems, AI models for early gastric cancer detection require formal evaluation and regulatory clearance from authorities such as the FDA or CE bodies (Singla et al., 2025). Such approval usually depends on robust external and prospective validation, which remains limited in current studies. From a methodological perspective, future improvements in DL-based EGC detection may benefit from incorporating Transformer-based architectures (e.g., Vision Transformer and Swin Transformer), which have shown strong performance in medical image analysis by capturing long-range spatial dependencies (Gandhi et al., 2025a). In addition, generative data augmentation techniques could help mitigate data imbalance and enhance model robustness (Gandhi et al., 2025b). The integration of multimodal learning frameworks, combining endoscopic video data with relevant clinical information, may further improve diagnostic accuracy and clinical relevance (Qin et al., 2025). To address data privacy and enhance generalizability, federated learning offers a promising strategy for leveraging multicenter data without direct data sharing (Assaf et al., 2025). Finally, adoption of standardized reporting guidelines, such as CONSORT-AI, DECIDE-AI, and STARD-AI, is essential to improve transparency, reproducibility, and clinical interpretability of future studies (Goh et al., 2025).

In addition, it is important to note that the generalizability of these performance estimates may be further challenged by the lack of temporal validation in most included studies. Robust clinical prediction systems require testing on data from future time periods to ensure stability against shifts in clinical practice, equipment, or patient demographics. In our review, only one study employed a prospective external validation set (Sakai et al., 2018). Future studies should prioritize this design to provide a more rigorous and clinically realistic assessment of model performance over time. Furthermore, the presence of publication bias in the external validation dataset likely stems from the limited number of available studies and potential selective reporting of higher-performing models in externally validated literature. The observed bias suggests that the overall diagnostic performance of DL models in external validation settings may be overestimated in the current literature. Therefore, the establishment of multi-center, large-scale external validation cohorts is essential for a comprehensive evaluation of DL model performance.

Several limitations of this meta-analysis should be acknowledged when interpreting the findings.

First, a fundamental limitation of this analysis is that all included studies utilized retrospective datasets for both model development and validation. This retrospective design inherently carries risks of selection bias and spectrum bias, where the case mix may not fully represent the broader clinical population encountered in practice. Therefore, while our meta-analysis suggests promising diagnostic potential, the reported high accuracy likely represents a “best-case” scenario. Forthcoming multi-center, prospective trials are crucial to rigorously evaluate model performance in unselected, consecutive patients under real-world conditions (Tong et al., 2023). Second, there was variability in the definition of EGC across the included studies, and the inclusion criteria for control groups were inconsistent. This heterogeneity in the control population—ranging from purely normal mucosa to a mix of benign lesions (e.g., gastric ulcers, low-grade epithelial neoplasia, gastric polyps)—constitutes a potential source of classification bias. Such inconsistency may lead to systematic differences in model training and evaluation, as models trained against purely normal mucosa might achieve higher specificity in distinguishing cancer from normal tissue but potentially lower sensitivity for discriminating early cancer from challenging benign or precancerous conditions. Third, the current analysis was restricted to image-level evaluation of DL models due to incomplete patient-level data in the included studies. However, patient-level assessment better aligns with clinical practice. Image-based training risks overfitting to specific features within individual patients, which may limit the model’s applicability to external datasets (Lengerich et al., 2018). Fourth, all included studies focused on detecting gastric lesions from static, high-quality white-light endoscopic images, which inherently cannot reproduce the complexity of real-time endoscopy. In actual clinical practice, endoscopic observation is dynamic and often affected by motion blur caused by scope movement, variations in illumination, changes in viewing angle, and transient interference from mucus, blood, bubbles, or food residue. These *in situ* factors substantially increase diagnostic difficulty but were largely excluded from the training and validation datasets of the included studies. Consequently, the reported diagnostic performance of AI models derived from idealized image datasets may overestimate their effectiveness in real-world, real-time clinical settings. Future studies should therefore prioritize validation using video-based or real-time endoscopic data that better reflect routine clinical conditions.

In conclusion, our meta-analysis provides robust evidence that DL algorithms exhibit high diagnostic efficacy in detecting EGC from white-light endoscopic images. Moreover, the sensitivity and specificity of these algorithms are comparable to those of expert endoscopists. These findings highlight the potential for DL algorithms to serve as a clinical decision-support tool in routine practice.

## Data Availability

The original contributions presented in the study are included in the article/Supplementary material, further inquiries can be directed to the corresponding author.
