# Supplementary material for: Deep learning for detecting early gastric cancer with white-light endoscopy: a systematic review and meta-analysis
Source: Front Artif Intell. 2026 Jan 29;9:1734591. doi: 10.3389/frai.2026.1734591 (PMC12894240; doi:10.3389/frai.2026.1734591)
Supplement: Supplementary file 1 [file Supplementary_file_1.docx]

**Supplementary Table 1** Preferred Reporting Items for Systematic Reviews and Meta-Analyses of Diagnostic Test Accuracy (PRISMA-DTA) Checklist.

| **Section/topic** | **#** | **PRISMA-DTA Checklist Item** | **Reported on page #** |
| --- | --- | --- | --- |
| **TITLE / ABSTRACT** | | |  |
| Title | 1 | Identify the report as a systematic review (+/- meta-analysis) of diagnostic test accuracy (DTA) studies. | 1 |
| Abstract | 2 | Abstract: See PRISMA-DTA for abstracts. | 1-2 |
| **INTRODUCTION** | | |  |
| Rationale | 3 | Describe the rationale for the review in the context of what is already known. | 3-4 |
| Clinical role of index test | D1 | State the scientific and clinical background, including the intended use and clinical role of the index test, and if applicable, the rationale for minimally acceptable test accuracy (or minimum difference in accuracy for comparative design). | 3-4 |
| Objectives | 4 | Provide an explicit statement of question(s) being addressed in terms of participants, index test(s), and target condition(s). | 4 |
| **METHODS** | | |  |
| Protocol and registration | 5 | Indicate if a review protocol exists, if and where it can be accessed (e.g., Web address), and, if available, provide registration information including registration number. | 4 |
| Eligibility criteria | 6 | Specify study characteristics (participants, setting, index test(s), reference standard(s), target condition(s), and study design) and report characteristics (e.g., years considered, language, publication status) used as criteria for eligibility, giving rationale. | 5 |
| Information sources | 7 | Describe all information sources (e.g., databases with dates of coverage, contact with study authors to identify additional studies) in the search and date last searched. | 4-5 |
| Search | 8 | Present full search strategies for all electronic databases and other sources searched, including any limits used, such that they could be repeated. | 4-5 |
| Study selection | 9 | State the process for selecting studies (i.e., screening, eligibility, included in systematic review, and, if applicable, included in the meta-analysis). | 4-5 |
| Data collection process | 10 | Describe method of data extraction from reports (e.g., piloted forms, independently, in duplicate) and any processes for obtaining and confirming data from investigators. | 6 |
| Definitions for data extraction | 11 | Provide definitions used in data extraction and classifications of target condition(s), index test(s), reference standard(s) and other characteristics (e.g. study design, clinical setting). | 4-5 |
| Risk of bias and applicability | 12 | Describe methods used for assessing risk of bias in individual studies and concerns regarding the applicability to the review question. | 6 |
| Diagnostic accuracy measures | 13 | State the principal diagnostic accuracy measure(s) reported (e.g. sensitivity, specificity) and state the unit of assessment (e.g. per-patient, per-lesion). | 6-7 |
| Synthesis of results | 14 | Describe methods of handling data, combining results of studies and describing variability between studies. This could include, but is not limited to: a) handling of multiple definitions of target condition. b) handling of multiple thresholds of test positivity, c) handling multiple index test readers, d) handling of indeterminate test results, e) grouping and comparing tests, f) handling of different reference standards | 7 |
| Meta-analysis | D2 | Report the statistical methods used for meta-analyses, if performed. | 7 |
| Additional analyses | 16 | Describe methods of additional analyses (e.g., sensitivity or subgroup analyses, meta-regression), if done, indicating which were pre-specified. | 7 |
| **RESULTS** | | |  |
| Study selection | 17 | Provide numbers of studies screened, assessed for eligibility, included in the review (and included in meta-analysis, if applicable) with reasons for exclusions at each stage, ideally with a flow diagram. | 8 |
| Study characteristics | 18 | For each included study provide citations and present key characteristics including: a) participant characteristics (presentation, prior testing), b) clinical setting, c) study design, d) target condition definition, e) index test, f) reference standard, g) sample size, h) funding sources | 8-10 |
| Risk of bias and applicability | 19 | Present evaluation of risk of bias and concerns regarding applicability for each study. | 10-11 |
| Results of individual studies | 20 | For each analysis in each study (e.g. unique combination of index test, reference standard, and positivity threshold) report 2x2 data (TP, FP, FN, TN) with estimates of diagnostic accuracy and confidence intervals, ideally with a forest or receiver operator characteristic (ROC) plot. | 11-15 |
| Synthesis of results | 21 | Describe test accuracy, including variability; if meta-analysis was done, include results and confidence intervals. | 11-15 |
| Additional analysis | 23 | Give results of additional analyses, if done (e.g., sensitivity or subgroup analyses, meta-regression; analysis of index test: failure rates, proportion of inconclusive results, adverse events). | 15 |
| **DISCUSSION** |  |  |  |
| Summary of evidence | 24 | Summarize the main findings including the strength of evidence. | 15-19 |
| Limitations | 25 | Discuss limitations from included studies (e.g. risk of bias and concerns regarding applicability) and from the review process (e.g. incomplete retrieval of identified research). | 19 |
| Conclusions | 26 | Provide a general interpretation of the results in the context of other evidence. Discuss implications for future research and clinical practice (e.g. the intended use and clinical role of the index test). | 19-20 |
| **FUNDING** |  |  |  |
| Funding | 27 | For the systematic review, describe the sources of funding and other support and the role of the funders. | 20 |

*Adapted From:*  McInnes MDF, Moher D, Thombs BD, McGrath TA, Bossuyt PM, The PRISMA-DTA Group (2018). Preferred Reporting Items for a Systematic Review and Meta-analysis of Diagnostic Test Accuracy Studies: The PRISMA-DTA Statement. JAMA. 2018 Jan 23;319(4):388-396. doi: 10.1001/jama.2017.19163.

**Supplementary Table 2** Search strategy in PubMed, Embase, Cochrane and Web of Science.

| Database | Search strategy |
| --- | --- |
| Pubmed | (((("Endoscopes"[Mesh] OR "Endoscopes, Gastrointestinal"[Mesh]) OR (((((((Endoscope[Title/Abstract]) OR (Endoscope, Gastrointestinal[Title/Abstract])) OR (Gastrointestinal Endoscope[Title/Abstract])) OR (Gastrointestinal Endoscopes[Title/Abstract])) OR (Endoscope[Title/Abstract]))) OR (((((("Gastroscopy"[Mesh]) OR (Gastroscopies[Title/Abstract])) OR (Gastroscopic Surgical Procedures[Title/Abstract])) OR (Gastroscopic Surgical Procedure[Title/Abstract])) OR (Gastroscopic Surgery[Title/Abstract])) OR (Gastroscopic Surgeries[Title/Abstract]))) AND (((("Machine Learning"[Mesh]) OR (Transfer Learning[Title/Abstract])) OR (((((((("Artificial Intelligence"[Mesh]) OR (Computer Reasoning[Title/Abstract])) OR (AI (Artificial Intelligence[Title/Abstract]))) OR (Machine Intelligence[Title/Abstract])) OR (Computational Intelligence[Title/Abstract])) OR (Computer Vision System[Title/Abstract])) OR (Knowledge Acquisition (Computer[Title/Abstract]))) OR (Knowledge Representation (Computer[Title/Abstract])))) OR (("Deep Learning"[Mesh]) OR (Hierarchical Learning[Title/Abstract])))) AND ((((((((("Stomach Neoplasms"[Mesh]) OR (Stomach Neoplasm[Title/Abstract])) OR (Gastric Neoplasms[Title/Abstract])) OR (Gastric Neoplasm[Title/Abstract])) OR (Cancer of Stomach[Title/Abstract])) OR (Stomach Cancer[Title/Abstract])) OR (Cancer of the Stomach[Title/Abstract])) OR (Gastric Cancer[Title/Abstract])) OR (Gastric Cancer, Familial Diffuse[Title/Abstract])) |
| Embase | ('artificial intelligence'/exp OR 'machine intelligence' OR 'artificial intelligence' OR 'machine learning'/exp OR 'learning machine' OR 'learning machines' OR 'machine learning' OR 'deep learning'/exp OR 'deep machine learning' OR 'deep ml' OR 'hierarchical learning' OR 'deep learning') AND ('stomach tumor'/exp OR 'gastric mass (tumor)' OR 'gastric masses (tumor)' OR 'gastric neoplasia' OR 'gastric neoplasm' OR 'gastric subepithelial tumor' OR 'gastric tumor' OR 'gastric tumorigenesis' OR 'gastric tumour' OR 'mucosa tumor, stomach' OR 'mucosa tumour, stomach' OR 'neoplasia of the stomach' OR 'neoplasm of the stomach' OR 'neoplasms of the stomach' OR 'neoplastic gastric' OR 'neoplastic stomach' OR 'stomach mucosa tumor' OR 'stomach mucosa tumour' OR 'stomach neoplasia' OR 'stomach neoplasm' OR 'stomach neoplasms' OR 'stomach tumorigenesis' OR 'stomach tumour' OR 'stomach ulcerated tumor' OR 'stomach ulcerated tumour' OR 'stomach ulcerating tumor' OR 'stomach ulcerating tumour' OR 'tumor of the gastric' OR 'tumor of the stomach' OR 'tumor, stomach mucosa' OR 'tumour of the gastric' OR 'tumour of the stomach' OR 'tumour, stomach mucosa' OR 'stomach tumor') AND ('endoscope'/exp OR 'ei-530b' OR 'en-450p5' OR 'en-450p5 standard' OR 'endocyto' OR 'endoscope workstation' OR 'endoscopes' OR 'evis exera iii' OR 'evis lucera elite' OR 'evis lucera spectrum' OR 'evis x1' OR 'gif h260z' OR 'gif-h260z' OR 'h260z' OR 'laduscope t flex pd-hs-0250' OR 'magnifying endoscope' OR 'r-scope' OR 'serpent (endoscope)' OR 'xillix onco life' OR 'endoscope' OR 'gastroscopy'/exp OR 'cardioendoscopy' OR 'endoscopy, cardia' OR 'fiber gastroscopy' OR 'fiber optic gastroscopy' OR 'fibergastroscopy' OR 'fibrogastroscopy' OR 'gastrofibroscopy' OR 'pylorobulboscopy' OR 'stomach endoscopy' OR 'gastroscopy') |
| Cochrane | #1 MeSH descriptor: [Stomach Neoplasms] explode all trees  #2 (Stomach Neoplasm):ti,ab,kw OR (Gastric Neoplasms):ti,ab,kw OR (Gastric Neoplasm):ti,ab,kw OR (Cancer of Stomach):ti,ab,kw OR (Stomach Cancer):ti,ab,kw OR (Cancer of the Stomach):ti,ab,kw OR (Gastric Cancer):ti,ab,kw OR (Gastric Cancer, Familial Diffuse):ti,ab,kw  #3 #1 OR #2  #4 MeSH descriptor: [Endoscopes] explode all trees  #5 MeSH descriptor: [Gastroscopy] explode all trees  #6 MeSH descriptor: [Endoscopes, Gastrointestinal] explode all trees  #7 (Endoscope):ti,ab,kw OR (Endoscope, Gastrointestinal):ti,ab,kw OR (Gastrointestinal Endoscope):ti,ab,kw OR (Gastrointestinal Endoscopes):ti,ab,kw OR (Endoscope):ti,ab,kw OR (Gastroscopies):ti,ab,kw OR (Gastroscopic Surgical Procedures):ti,ab,kw OR (Gastroscopic Surgical Procedure):ti,ab,kw OR (Gastroscopic Surgery):ti,ab,kw OR (Gastroscopic Surgeries):ti,ab,kw  #8 #4 OR #5 OR #6 OR #7  #9 MeSH descriptor: [Machine Learning] explode all trees  #10 MeSH descriptor: [Artificial Intelligence] explode all trees  #11 MeSH descriptor: [Deep Learning] explode all trees  #12 (Transfer Learning):ti,ab,kw OR (Computer Reasoning):ti,ab,kw OR (AI (Artificial Intelligence)):ti,ab,kw OR (Machine Intelligence):ti,ab,kw OR (Computational Intelligence):ti,ab,kw OR (Computer Vision System):ti,ab,kw OR (Knowledge Acquisition (Computer)):ti,ab,kw OR (Knowledge Representation (Computer)):ti,ab,kw OR (Hierarchical Learning):ti,ab,kw  #13 #9 OR #10 OR #11 OR #12  #14 #3 AND #8 AND #13 |
| Web of science | TS=(Stomach Neoplasms OR Stomach Neoplasm OR Gastric Neoplasm OR Cancer of Stomach OR Cancer of the Stomach OR Gastric Cancer OR Stomach Cancer) AND TS=(Artificial Intelligence OR Machine learning OR Deep Learning OR Computer Reasoning OR AI Artificial Intelligence OR Machine Intelligence OR Computational Intelligence OR Computer Vision System OR Knowledge Acquisition (Computer) OR Knowledge Representation (Computer) OR Transfer Learning OR Hierarchical Learning) AND TS=(Endoscopes OR Gastroscopy OR Endoscope OR Gastrointestinal Endoscope OR Gastrointestinal Endoscopes OR gastroscopes OR gastroscopica Surgical Procedures OR gastroscopica Surgical Procedure OR gastroscopica Surgery OR gastroscopica Surgeries) |

**Supplementary Table 3** Characteristics of control groups and endoscopic equipment in included studies.

| Study (Year) | Control Group Composition | Endoscope Brand | Endoscope Model |
| --- | --- | --- | --- |
| Sakai et al. (2018) | Normal subjects | Olympus | GIF-H290Z |
| Cho et al. (2019) | Patients with advanced gastric cancer, low-grade or high-grade intraepithelial neoplasia, and non-neoplastic lesions | Olympus | GIF-Q260, GIF-H260, GIF-H290 |
| Tang et al. (2020) | Non-atrophic or atrophic gastritis patients, with or without ulceration | Olympus | GIF-H260, GIF-HQ290 |
| Zhang et al. (2021) | Patients with peptic ulcer, advanced gastric cancer, gastric submucosal tumor, and normal subjects | Olympus | GIF-H260, GIF-Q260J, GIF-H290 |
| Zhou et al. (2022) | Patients with early gastric cancer | Olympus,Fujifilm | GIF-Q260J, GIF-H260Z, GIF-HQ290, GIF-H290Z, EG-L590ZW, EG-L600ZW |
| Yuan et al. (2022) | Patients with advanced gastric cancer, submucosal tumor, polyp, peptic ulcer, or erosion, and normal subjects | Olympus | GIF-Q260, GIF-H260, GIFH290 |
| Teramoto et al. (2022) | Patients with advanced gastric cancer and normal subjects | Olympus,Fujifilm | IF-290Z, GIFHQ290, GIF-XP290N, GIF-260Z, EG-L600ZW7 |
| Takemoto et al. (2023) | Patients with early gastric cancer | Olympus | GIF-H290Z, GIFH260Z |
| Gong et al. (2023) | Patients with gastric mucosal atrophy, intestinal metaplasia, dysplasia, advanced gastric cancer and normal subjects | Olympus | GIF-Q260, GIF-H260, GIF-H290 |
| Dong et al. (2023) | Patients with gastric mucosal atrophy, intestinal metaplasia, chronic inflammation, or polyps | Not mentioned | Not mentioned |
| Zhang et al. (2023) | Gastric and duodenal diseases: xanthoma, polyp, ulcer, fundic gland tumor, erosion, intraepithelial neoplasia, and other structural anomalies.  Esophageal diseases: Barrett’s esophagus, reflux esophagitis, ectopic mucosa, erosion, and other mucosal lesions | Not mentioned | Not mentioned |
| Zhang et al. (2024) | Normal subjects | Not mentioned | Not mentioned |
| Chang et al. (2024) | Patients with intraepithelial neoplasia, erosion, ulcer, or polyps | Olympus | GIF-Q260, GIF-H260, GIF-H290 |
| Haq et al. (2024) | Patients with advanced gastric cancer and normal subjects | Olympus | GIF-H260Z, GIF-H290Z |
| Feng et al. (2025) | Patients with gastric mucosal ulcer or chronic inflammation, and normal subjects | Olympus | GIF-H260, GIF-H260Z, GIF-HQ290, GIFH290Z |

**Supplementary Table 4**. Comparison of diagnostic performance between artificial intelligence and endoscopists

| Author | Year | Artificial intelligence model | | | |  | Endoscopist | | | |
| --- | --- | --- | --- | --- | --- | --- | --- | --- | --- | --- |
|  |  | TP^a^ | FP^b^ | TN^c^ | FN^d^ |  | TP | FP | TN | FN |
| Cho et al. | 2019 | 13 | 33 | 136 | 18 |  | 26 | 16 | 138 | 20 |
| Tang et al. | 2020 | 279 | 7 | 293 | 21 |  | 248 | 24 | 276 | 52 |
| Zhang et al. | 2021 | 92 | 74 | 766 | 158 |  | 80 | 112 | 729 | 170 |
| Yuan et al. | 2022 | 177 | 146 | 1247 | 9 |  | 209 | 34 | 1222 | 114 |
| Takemoto et al. | 2023 | 27 | 21 | 320 | 11 |  | 19 | 4 | 337 | 19 |

^a^TP: true positive.

^b^TN: true negative.

^c^FP: false positive.

^d^FN: false negative

**Supplementary Table 5** Leave-one-out sensitivity analysis for sensitivity and specificity of internal validation sets

| Omitting study | Sensitivity (95%CI) | I2 for sensitivity (%) | Specificity (95%CI) | I2 for specificity (%) |
| --- | --- | --- | --- | --- |
| Sakai et al. | 0.91 (0.82 - 0.96) | 99.41 | 0.93 (0.86 - 0.97) | 98.96 |
| Cho et al. | 0.88 (0.87 - 0.89) | 98.9 | 0.90 (0.90 - 0.91) | 99.0 |
| Tang et al. | 0.90 (0.80 - 0.95) | 98.88 | 0.94 (0.87 - 0.97) | 98.78 |
| Zhang et al.(2021) | 0.92 (0.86 - 0.96) | 99.15 | 0.93 (0.86 - 0.97) | 99.24 |
| Zhou et al. | 0.93 (0.93 - 0.93) | 99.50 | 0.12 (0.12 - 0.13) | 99.00 |
| Yuan et al. | 0.90 (0.81 - 0.95) | 99.39 | 0.93 (0.87 - 0.97) | 99.22 |
| Teramoto et al. | 0.88 (0.81 - 0.93) | 99.09 | 0.91 (0.87 - 0.93) | 98.58 |
| Dong et al. | 0.91 (0.81 - 0.96) | 99.42 | 0.94 (0.88 - 0.97) | 99.24 |
| Takemoto et al. | 0.91 (0.82 - 0.96) | 99.41 | 0.94 (0.87 - 0.97) | 99.23 |
| Gong et al. | 0.93 (0.92 - 0.93) | 99.50 | 0.12 (0.12 - 0.13) | 99.00 |
| Zhang et al.(2023) | 0.91 (0.81 - 0.96) | 99.40 | 0.94 (0.87 - 0.97) | 99.20 |
| Zhang et al.(2024) | 0.90 (0.80 - 0.95) | 99.35 | 0.93 (0.86 - 0.97) | 99.17 |
| Haq et al. | 0.90 (0.80 - 0.95) | 99.29 | 0.93 (0.85 - 0.96) | 99.06 |
| Chang et al. | 0.90 (0.81 - 0.95) | 99.35 | 0.93 (0.86 - 0.97) | 99.16 |
| Feng et al. | 0.90 (0.81 - 0.96) | 99.37 | 0.93 (0.86 - 0.97) | 99.21 |


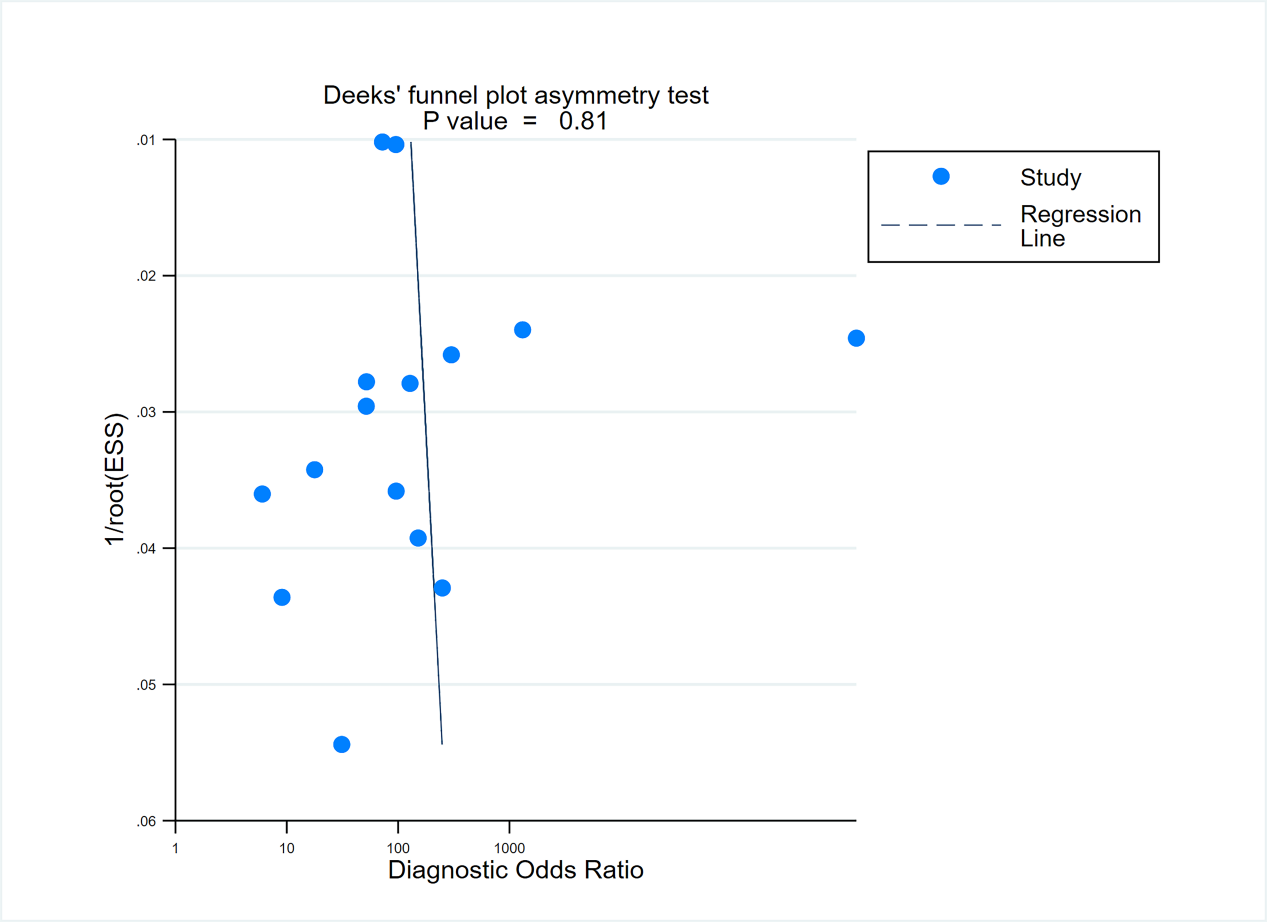


**Supplementary Fig. 1** Deeks’ funnel plot for assessing asymmetry in the diagnostic performance of deep learning algorithms for detecting early gastric cancer (EGC) in the internal validation set.


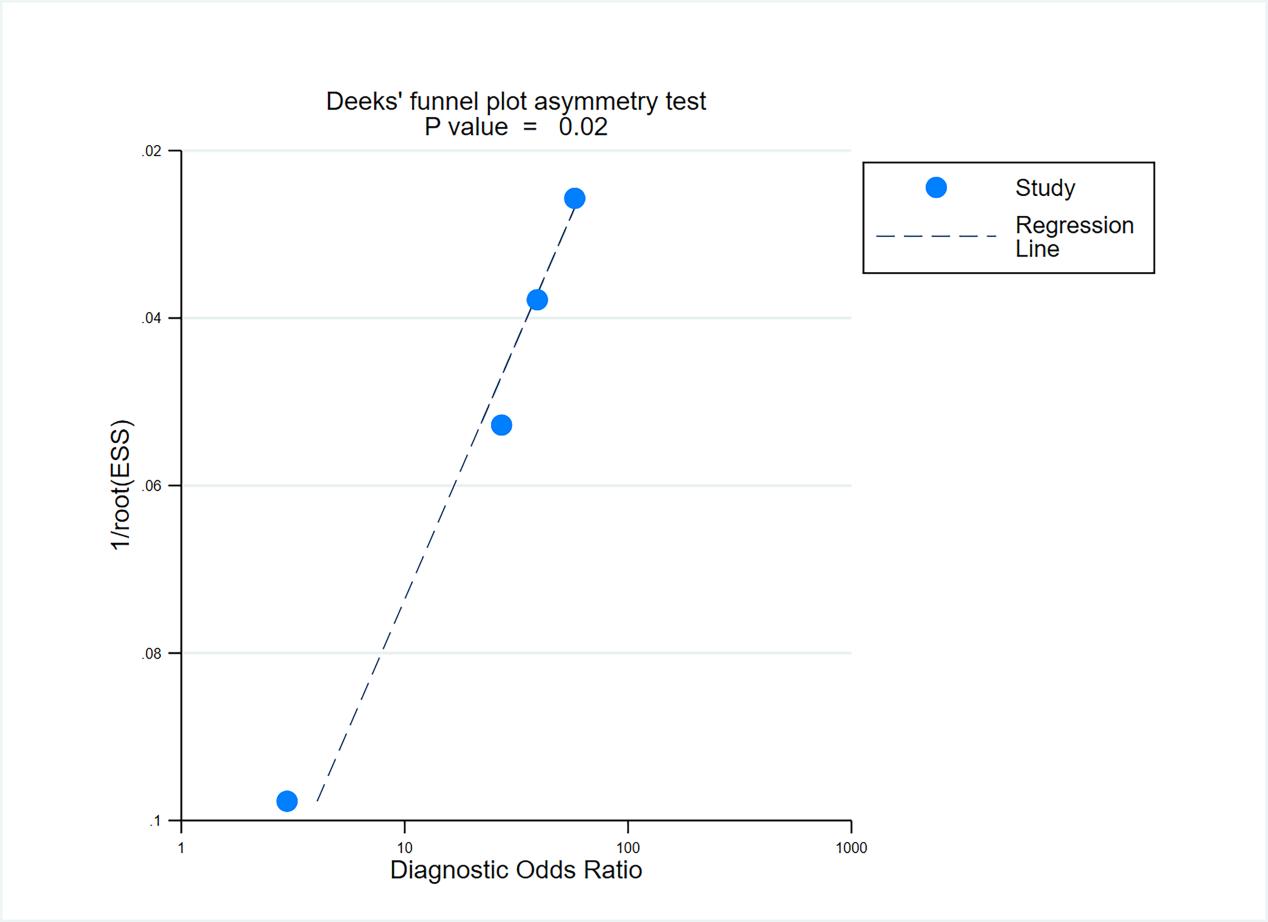


**Supplementary Fig. 2** Deeks’ funnel plot for assessing asymmetry in the diagnostic performance of deep learning algorithms for detecting early gastric cancer (EGC) in the external validation set.
